# Impact of artificial intelligence support on accuracy and reading time in breast tomosynthesis image interpretation: a multi-reader multi-case study

**DOI:** 10.1007/s00330-021-07992-w

**Published:** 2021-05-04

**Authors:** Suzanne L. van Winkel, Alejandro Rodríguez-Ruiz, Linda Appelman, Albert Gubern-Mérida, Nico Karssemeijer, Jonas Teuwen, Alexander J. T. Wanders, Ioannis Sechopoulos, Ritse M. Mann

**Affiliations:** 1 Department of Medical Imaging, Radboud University Medical Center, PO Box 9101, 6500 HB Nijmegen, Geert Grooteplein 10, 6525 GA, Post 766 Nijmegen, The Netherlands; 2 ScreenPoint Medical BV, Toernooiveld 300, 6525 EC Nijmegen, The Netherlands; 3 Department of Radiation Oncology, Netherlands Cancer Institute (NKI), Plesmanlaan 121, 1066 CX Amsterdam, The Netherlands; 4 Bevolkingsonderzoek Zuid-West Borstkanker, Laan 20, 2512 GB Den Haag, The Netherlands; 5 Dutch Expert Centre for Screening (LRCB), Wijchenseweg 101, 6538 SW Nijmegen, The Netherlands; 6 Department of Radiology, Netherlands Cancer Institute (NKI), Plesmanlaan 121, 1066 CX Amsterdam, The Netherlands

**Keywords:** Digital breast tomosynthesis (DBT), Artificial intelligence (AI), Breast cancer, Mammography, Mass screening

## Abstract

**Objectives:**

Digital breast tomosynthesis (DBT) increases sensitivity of mammography and is increasingly implemented in breast cancer screening. However, the large volume of images increases the risk of reading errors and reading time. This study aims to investigate whether the accuracy of breast radiologists reading wide-angle DBT increases with the aid of an artificial intelligence (AI) support system. Also, the impact on reading time was assessed and the stand-alone performance of the AI system in the detection of malignancies was compared to the average radiologist.

**Methods:**

A multi-reader multi-case study was performed with 240 bilateral DBT exams (71 breasts with cancer lesions, 70 breasts with benign findings, 339 normal breasts). Exams were interpreted by 18 radiologists, with and without AI support, providing cancer suspicion scores per breast. Using AI support, radiologists were shown examination-based and region-based cancer likelihood scores. Area under the receiver operating characteristic curve (AUC) and reading time per exam were compared between reading conditions using mixed-models analysis of variance.

**Results:**

On average, the AUC was higher using AI support (0.863 vs 0.833; *p* = 0.0025). Using AI support, reading time per DBT exam was reduced (*p < *0.001) from 41 (95% CI = 39–42 s) to 36 s (95% CI = 35– 37 s). The AUC of the stand-alone AI system was non-inferior to the AUC of the average radiologist (+0.007, *p* = 0.8115).

**Conclusions:**

Radiologists improved their cancer detection and reduced reading time when evaluating DBT examinations using an AI reading support system.

**Key Points:**

*• Radiologists improved their cancer detection accuracy in digital breast tomosynthesis (DBT) when using an AI system for support, while simultaneously reducing reading time.*

*• The stand-alone breast cancer detection performance of an AI system is non-inferior to the average performance of radiologists for reading digital breast tomosynthesis exams.*

*• The use of an AI support system could make advanced and more reliable imaging techniques more accessible and could allow for more cost-effective breast screening programs with DBT.*

## Introduction

In recent years, several clinical trials have demonstrated how using digital breast tomosynthesis (DBT) as a breast cancer screening modality may improve screening results compared to 2D mammography, leading to increased cancer detection and a reduction of recalls [1–3]. Albeit a reduction in the frequency of interval cancers has not yet been shown [4], the improved detection is possible because DBT generates a pseudo-3D volume of the breast which partially overcomes one of the main limitations of any 2D imaging technique: tissue superposition [5]. However, the introduction of DBT as a screening modality still faces difficulties. The interpretation of DBT screening exams takes significantly longer compared to interpreting 2D mammography images [6, 7]. Particularly in settings where exams are double-read, like most European screening programs, the increasing lack of specialized breast radiologists [8] reduces the potential of DBT introduction. Deep learning–based artificial intelligence (AI) systems are quickly gaining attention in the field of radiology, particular in breast imaging [9]. The current stand-alone performance of AI systems for mammography is approaching, if not already exceeding, the performance of radiologists [10–13]. This may result in tools that sustain current mammography-based breast cancer screening programs with less human interaction or even improve the overall quality of screening [14, 15]. AI support in screening with DBT could improve cost-efficiency by increasing radiologists’ breast cancer detection performance, allowing radiologists to read DBT exams faster [16, 17], or triaging the studies [15].

The first studies investigating the impact of using AI during DBT interpretation use narrow-angle DBT examinations (with scan angle of 20° or lower) [16]. However, technical specifications of DBT are highly variable across vendors of DBT systems, leading to more substantial differences in the resulting images compared to mammography [18]. This is mainly due to differences in the angular range of the various machine models, the reconstruction, and other post-processing algorithms. Technically, a wider angle provides a higher depth resolution [18] and may enable better separation of lesions from superimposed fibroglandular tissue, but may lead to a poorer calcification depiction [5].

This study evaluates the impact of an AI support system in wide-angle DBT, previously validated for 2D mammograms [10, 11, 15], on radiologists’ accuracy and reading time. It was hypothesized that radiologists’ average performance in the detection of malignancies using AI support is superior to reading unaided. In addition, the aim was to demonstrate whether AI support could improve radiologists’ average reading time while maintaining or improving sensitivity and specificity and to compare the stand-alone detection performance of the AI system to the average radiologist.

## Materials and methods

A HIPAA-compliant fully-crossed fully-randomized multi-reader multi-case (MRMC) study was performed with 18 radiologists, reading a series of wide-angle DBT exams twice, with and without AI support.

### Study population

#### Case collection

This study included 360 cases: 110 biopsy-proven cancer cases, 104 benign cases (proven by biopsy or at least 6-month follow-up), and 146 randomly selected negative cases (at least 1-year follow-up). Cases were collected from a dataset of a previous, IRB-approved, clinical trial registered with protocol number NCT01373671 [19, 20]. Data was collected between May 2011 and February 2014 from seven US clinical sites, representative of women undergoing screening and diagnostic DBT exams in the USA. The mean age was 56.3 ± 9.8 (standard deviation) years. Each case consists of a bilateral two-view (cranio-caudal/mediolateral oblique CC/MLO) DBT exam acquired using standard exposure settings with a Mammomat Inspiration (Siemens Healthineers)) and reconstructed with the latest algorithm (EMPIRE), also generating the corresponding synthetic mammography (SM) images [21]. The DBT system has a wide 50° scanning angle. This data was not used for the development of the AI support system.

#### Case selection protocol

The case selection was aimed at obtaining a challenging and representative set for the observer evaluation. Exclusion criteria were as follows: breast implants, sub-optimal quality (judged by a radiologist and a radiographer with respectively 14 and 38 years of breast imaging experience), missing image data, or missing truth data. After exclusion and performing a power analysis [22], to achieve a power of at least 0.8 (80%) to test the primary hypothesis of the study, we target to select 110 negative cases, 65 benign cases, and 65 malignant cases. Negative and benign cases were randomly selected to avoid selection bias. The aim for the malignant case selection was to include all cases categorized as “subtle”, and as many “moderately subtle” cases as available while including at least a random selection of five “obvious” cases. To reach the targeted sample size of 65 malignant cases, a subtlety score (1, “subtle”; 2, “moderately subtle”; 3, “obvious”) was independently determined by three breast radiologists (respectively 14, 39, and 5 years mammography experience; 5, 5, and 5 years DBT experience), with the third acting as an arbiter in case of disagreement.

#### Reference standard

For every case, per breast, the reference standard based on pathology and imaging reports was available in electronic format and reviewed by the radiologists participating in the case selection process (not participating in the observer study), including location and radiological characterization of cancers, location of benign lesions, or confirmed normal status.

### AI support system

The AI support system used during the observer evaluation was Transpara™ 1.6.0, (ScreenPoint Medical BV). This system is based on deep convolutional neural networks [23, 24] and automatically detects lesions suspicious of breast cancer in 2D and DBT mammograms from different vendors. The results are shown to radiologists in two distinct ways:

- A score from 1 to 10, indicating the increasing likelihood that a visible cancer is present at the mammogram. In a screening setting, approximately 10% of mammograms are assigned each score.
- The most suspicious findings are marked and a scored with the level of suspicion (LOS) for cancer (1–100).

The system has been validated for 2D mammograms in previously performed clinical studies with independent datasets [10, 11, 15]. It has been trained and tested using a proprietary database containing over 1,000,000 2D mammography and DBT images (over 20,000 with cancer), acquired with machines from five different mammography vendors at a dozen institutions (academic and private health centers) across 10 countries in Europe, America, and Asia.

Each selected DBT mammogram was processed by the AI system. The results of this analysis were shown during the observer evaluation. Radiologists could concurrently use the AI system with or without the corresponding SM and interactive navigation support. Interactive navigation support consists of automatic access to the DBT plane where the AI algorithm detected abnormalities, with a single click on a mark shown on the SM.

### Observer evaluation

#### Sessions and training

The observer evaluation consisted of two parts. Exams were read twice, with and without AI support, separated by a wash-out period of at least 4 weeks. The case order and the availability of AI support were randomized for each radiologist.

During the evaluation of the cases with the AI support available, two reading protocols were tested. Half of the radiologists (readers 1–9) read the exams with access to the corresponding SM and interactive navigation support, while the other half (readers 10–18) read exams without these functionalities, showing AI findings only in the 3D DBT stack.

To get familiar with the AI system and workstation before participating in the study, all radiologists were trained by evaluating a set of 50 DBT exams (not included in the study). Radiologists were blinded to patient history and any other information not visible in the included DBT imaging exams.

#### Reporting

The radiologists used a reading workstation for DBT exams and a 12MP DBT-certified diagnostic color display (Coronis Uniti, Barco) calibrated to the DICOM Grayscale Standard Display Function. The workstation tracked the reader actions in the interface with timestamps.

For every case, radiologists were instructed to the following:

- Mark the 3D location of findings in every view visible
- Assign a LOS to each finding
- Provide a BI-RADS category (1, 2, 3, 4a, 4b, 4c, or 5) per breast.

#### Readers

All radiologists were American Board of Radiology–certified, qualified to interpret mammograms under the Mammography Quality Standard Act (MQSA) and active in reading DBT exams in clinical practice. Half of the readers devoted less than 75% of their professional time to breast imaging for the last 3 years while the other half devoted more time. The median experience with MQSA qualification of the readers was 9 years (range 2–23 years) and the median volume of 2D or DBT mammograms read per year was 4200 (1000-18,000). All the readers were at the time of the study reading DBT exams in clinical practice.

### Endpoints and statistical analysis

#### Primary hypothesis

The primary hypothesis was that radiologists’ average breast-level area under the receiver operating characteristic (ROC) curve (AUC) for detection of malignancies in DBT using AI reading support is superior to reading unaided. This was tested against the null hypothesis: radiologists’ average breast-level AUC with AI support being equivalent to their average AUC unaided. *p* < 0.05 indicated a statistically significant difference between both reading conditions.

AUC superiority analysis was performed using the statistical package developed by Tabata et al [25], using the Obuchowski and Rockette method adapted to consider clustered data when calculating reader by modality covariances [26, 27].

ROC curves were built using the LOS assigned to each breast. Standard errors (SE) and 95% confidence intervals (CI) were computed.

#### Secondary hypotheses

If the primary hypothesis was met, four secondary hypotheses were evaluated in a (hierarchical) fixed sequence to control type I error rate at significance level alpha = 0.05.

i. Radiologists’ average reading time per DBT exam using AI support is superior to (shorter than) the average reading time per DBT exam unaided.

Average reading times per DBT exam were compared between reading conditions by using a generalized linear mixed-effects (GLME) model, taking repeated measures by multiple readers into account [28].

ii. Radiologists’ average sensitivity reading DBT exams with AI support is non-inferior/superior compared to reading DBT exams unaided, at a pre-specified non-inferiority margin delta of 0.05.
iii. Radiologists’ average specificity reading DBT exams with AI support is non-inferior/superior compared to reading DBT exams unaided, at a pre-specified non-inferiority margin delta of 0.05.

The analysis was performed following the analysis described in the primary hypothesis for AUC comparisons, formatting the input data accordingly. A breast was considered positive if the radiologist assigned a BI-RADS score ≥ 3.

iv. The stand-alone AI system AUC is non-inferior to the radiologists’ average breast-level AUC reading DBT exams unaided at a pre-specified non-inferiority margin delta of 0.05.

The public domain iMRMC software (version 4.0.3, Division of Imaging, Diagnostics, and Software Reliability, OSEL/CDRH/FDA) was used, which can also handle single reader data (the AI system) [29].

## Results

### Study population

**Fig. 1:**
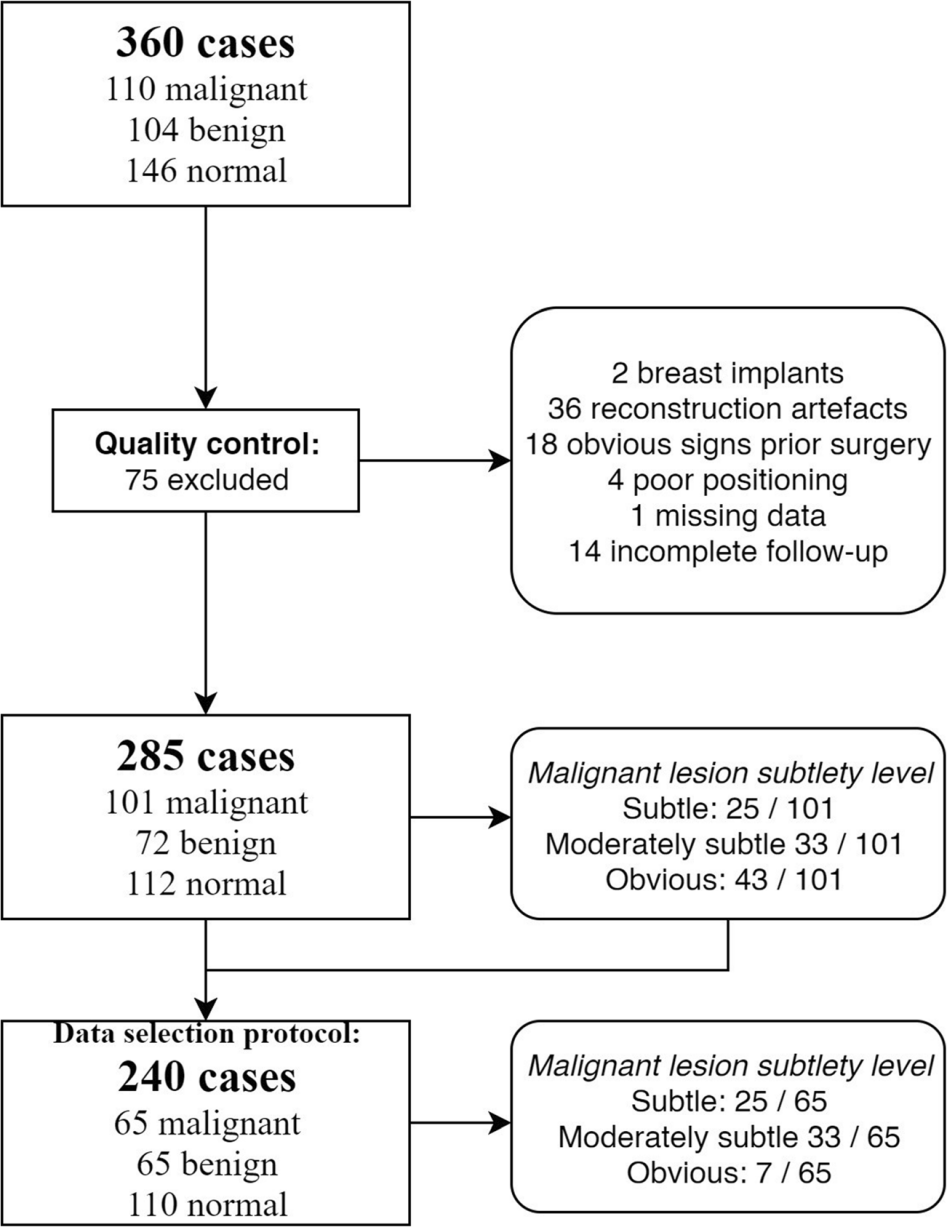
Flow of women through the study, from data collection until data selection for the observer evaluation

Figure 1 shows the case selection flowchart. The characteristics of the selected sample are detailed in Tables 1 and 2. No protocol deviations were found during data selection.

**Table 1:** Truth status on a case, breast, and lesion level of the cohort of 240 cases used in the observer evaluation

Truth	Cases	Breasts	Lesions
Normal	110/240 (46%)	334/480 (70%)	n/a
Benign*	65/240 (27%)	75/480 (16%)	86
Malignant**	65/240 (27%)	71/480 (14%)	85

*Within the benign breasts, six are the contralateral breast to a malignant breast. Two breasts containing a malignant and a benign lesion were regarded as malignant at the breast level. Four cases have benign lesions in both breasts. Seven breasts have two benign lesions and one breast has three benign lesions

**Six cases have breast cancer lesions in both breasts

**Table 2:** Characteristics of the cohort of 240 cases used in the observer evaluation, including the pathological and morphological characteristics of the lesions.

Cases	240
Mean age in years (range)	56 (30–81)
Median compressed breast thickness in mm (range)	59 (27–99)
BI-RADS breast density	A: 27/240 (11%) B: 93/240 (39%) C: 105/240 (44%) D: 15/240 (6%)
Median size in mm of malignant lesions (range)	15 (3–116)
Morphology of the key findings of malignant breasts	Mass: 40/71 (56%) Calcifications: 17/71 (24%) Mass + calcifications: 2/71 (3%) Architectural distortion: 8/71 (11%) Mass + architectural distortion: 1/71 (1%) Asymmetric density: 3/71 (4%)
Morphology of the key findings of benign breasts	Mass: 26/75 (35%) Calcifications: 38/75 (51%) Mass + calcifications: 2/75 (3%) Architectural distortion: 4/75 (5%) Asymmetric density: 4/75 (5%) Mass + asymmetric density: 1/75 (1%)
Histology of malignant breasts	IDC (invasive ductal ca.): 32/71 (45%) ILC (invasive lobular ca.): 7/71 (10%) DCIS (ductal ca. in situ): 15/71 (21%) IDC + DCIS: 11/71 (15%) IDC + other invasive type: 4/71 (6%) Invasive papilloma: 1/71 (1%) Invasive medullar ca.: 1/71 (1%)
Histology of benign breasts	Atypical ductal hyperplasia: 22/75 (29%) Fibroadenoma: 12/75 (16%) Adenosis: 6/75 (8%) Fibrocystic changes: 5/75 (7%) Breast cysts: 4/75 (5%) Fat necrosis: 3/75 (4%) Papilloma: 3/75 (4%) Apocrine metaplasia: 2/75 (3%) Others: 18/75 (24%)

### Impact on breast cancer detection accuracy

All readers completed the reading sessions as planned; 8640 case reports were received (240 × 18 × 2), and there was no missing data.

**Fig. 2:**
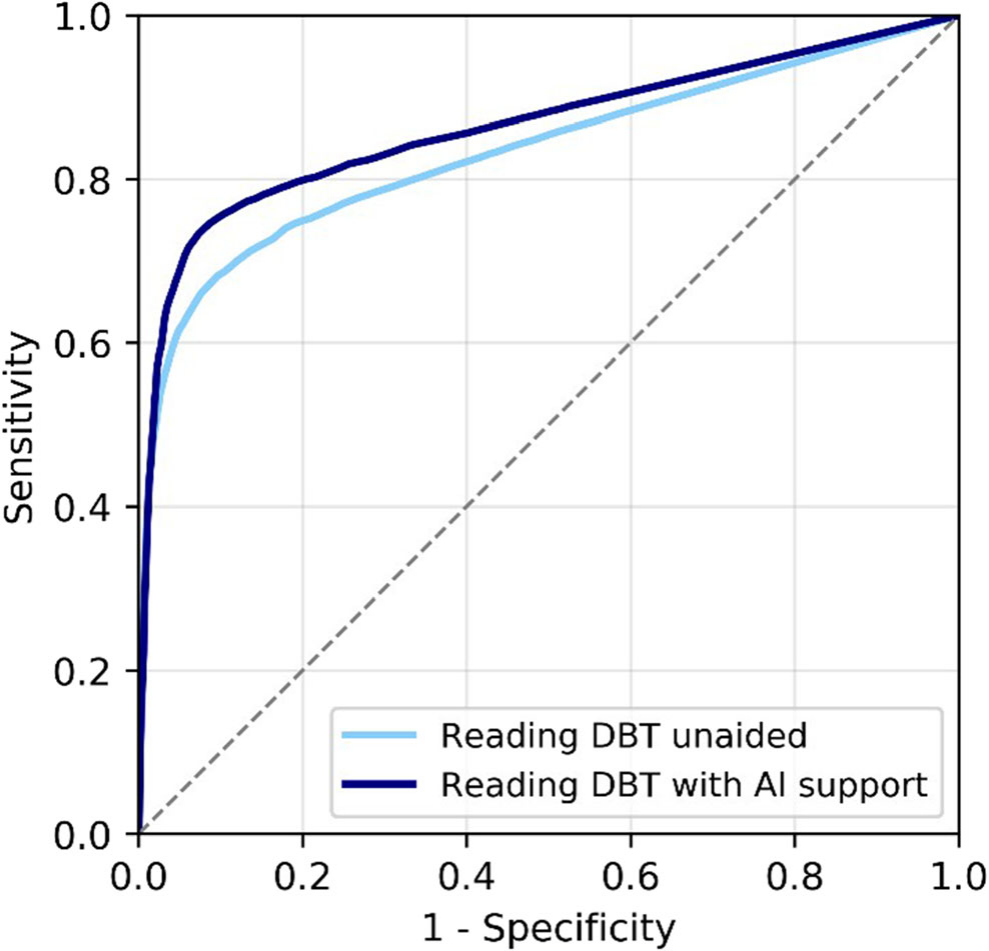
Average receiver operating characteristic curves (ROC) of the radiologists reading breast tomosynthesis (DBT) unaided and reading DBT exams with AI support concurrently. The difference in ROC area under the curve was significant, + 0.03, *p* = 0.0025

Radiologists significantly improved their DBT detection performance using AI support. The average AUC increased from 0.833 (95% CI = 0.799–0.867) to 0.863 (95% CI = 0.829–0.898), *p* = 0.0025 (difference + 0.030, 95% CI = 0.011–0.049). The average ROC curves are presented in Fig. 2. Differences per reader are shown in Table 3. Sixteen out of 18 readers (89%) had a higher AUC using AI support; improvements ranged from + 0.010 to + 0.088.

**Table 3:** Differences in the area under the receiver operating characteristic curve (AUC) and reading time for each radiologist between reading breast tomosynthesis unaided and reading breast tomosynthesis with AI support. *Rad*, radiologist; *SE*, standard error; *CI*, confidence interval. > 75% = in the last 3 years > 75% devoted to breast imaging

			AUC (SE)			Reading time in s (95% CI)		
Rad.	With SM	> 75%	Unaided	With AI support	Difference	Unaided	With AI support	% difference
1	Y	N	0.817 (0.031)	0.837 (0.029)	+ 0.020 (0.030)	45 (43, 48)	33 (31, 36)	−26 (−31, −21)
2	Y	N	0.745 (0.031)	0.792 (0.030)	+ 0.047 (0.034)	19 (17, 21)	16 (14, 18)	−17 (−29, −4)
3	Y	Y	0.813 (0.030)	0.901 (0.023)	+ 0.088 (0.030)	29 (27, 31)	27 (25, 29)	−6 (−14, 2)
4	Y	Y	0.862 (0.027)	0.872 (0.026)	+ 0.010 (0.027)	67 (64, 70)	48 (46, 55)	−28 (−32, −25)
5	Y	N	0.820 (0.032)	0.868 (0.028)	+ 0.047 (0.029)	62 (59, 64)	49 (46, 51)	−21 (−25, −17)
6	Y	Y	0.886 (0.027)	0.896 (0.025)	+ 0.011 (0.018)	46 (44, 49)	38 (35, 40)	−19 (−24, −14)
7	Y	N	0.799 (0.030)	0.863 (0.027)	+ 0.065 (0.031)	45 (43, 47)	26 (24, 28)	−42 (−48, −38)
8	Y	Y	0.837 (0.029)	0.884 (0.026)	+ 0.047 (0.030)	24 (22, 26)	28 (26, 30)	20 (10, 30)
9	Y	N	0.801 (0.031)	0.837 (0.027)	+ 0.036 (0.031)	27 (25, 29)	27 (25, 29)	2 (−7, 10)
10	N	Y	0.878 (0.026)	0.892 (0.024)	+ 0.014 (0.027)	67 (65, 70)	71 (68, 74)	6 (3, 9)
11	N	N	0.849 (0.029)	0.882 (0.026)	+ 0.032 (0.026)	55 (52, 57)	57 (54, 59)	4 (−1, 8)
12	N	N	0.835 (0.029)	0.829 (0.030)	-0.006 (0.025)	41 (38, 43)	37 (35, 40)	−7 (−13, −2)
13	N	Y	0.859 (0.027)	0.861 (0.027)	+ 0.014 (0.029)	40 (38, 43)	32 (30, 34)	−22 (−27, −16)
14	N	Y	0.873 (0.026)	0.850 (0.028)	-0.023 (0.031)	24 (21, 26)	15 (13, 17)	−3 (−47, −27)
15	N	N	0.849 (0.029)	0.889 (0.026)	+ 0.036 (0.028)	64 (62, 67)	61 (58, 63)	−6 (−9, −2)
16	N	N	0.780 (0.031)	0.835 (0.029)	+ 0.054 (0.031)	23 (21, 25)	31 (29, 34)	38 (28, 48)
17	N	N	0.796 (0.029)	0.849 (0.028)	+ 0.053 (0.029)	19 (17, 21)	23 (21, 25)	20 (7, 32)
18	N	Y	0.898 (0.025)	0.908 (0.023)	+ 0.010 (0.025)	54 (52, 57)	47 (44, 49)	−15 (−19, −10)

Descriptive analysis showed AUC improvements when using AI support were present in all subgroups:

- Lesion type (+ 0.022 [95% CI = −0.005, 0.049] for cases with soft tissue lesions, + 0.046 [95% CI = 0.015, 0.077] for cases with calcifications)
- Reading protocol (+ 0.041 [95% CI = 0.019, 0.063] for radiologists using SM and interactive navigation, + 0.020 [95% CI = −0.004, 0.044] for radiologists reading DBT alone)
- Radiologists’ specialization (+0.020 [95% CI = −0.007, 0.047] for radiologists who dedicated > 75% of their professional time to breast imaging in the last 3 years, +0.038 [95% CI = 0.022, 0.054] for the rest).

### Impact on reading time

The average reading time per DBT exam was significantly shorter using AI support (36 s, 95% CI = 35–37s) compared to reading unaided (41 s, 95% CI = 39–42s); resulting in a difference of −11% (95% CI = −8%, −13%), *p* < 0.001 (Table 3). Descriptively, reading time was shorter using AI support, regardless of breast density (low breast density: −13% (95% CI = −10%, −16%); high breast density: −10% (95% CI = −7%, −13%)) or reading protocol. However, the reduction was larger using 2D SM images and interactive navigation (shorter for 7/9 radiologists, decreasing from 39 to 32s, a difference of −19%, 95% CI = −16%, −22%), than without these tools (shorter for 5/9 radiologists (56%), decreasing from 42 to 40s, a difference of −4%, 95% CI = −1%, −7%).

**Fig. 3:**
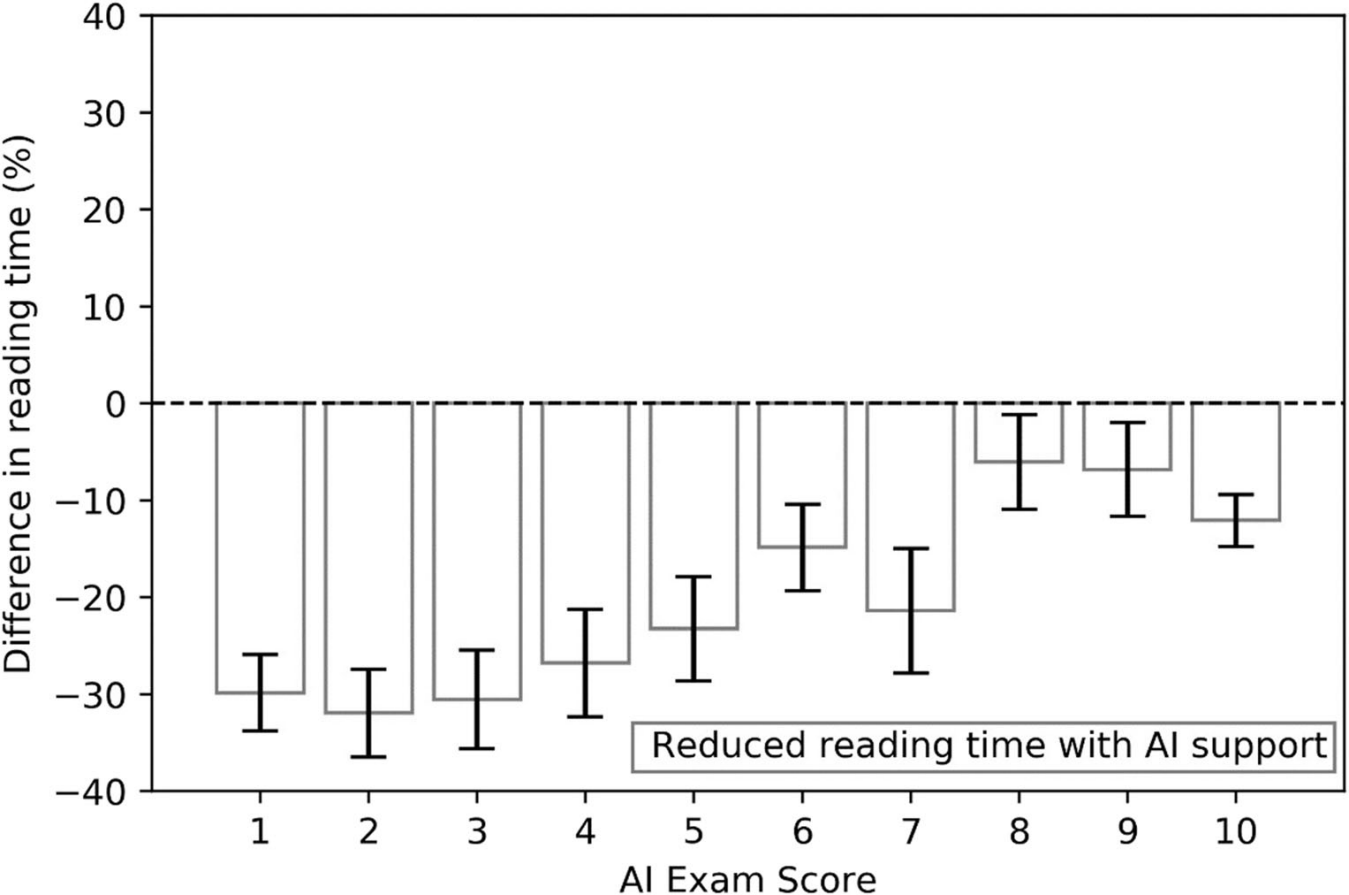
Average differences in reading time (%) across radiologists using synthetic mammograms and interactive navigation features between reading breast tomosynthesis exams unaided or reading with AI support, as a function of the exam-level score assigned by the AI system

**Fig. 4:**
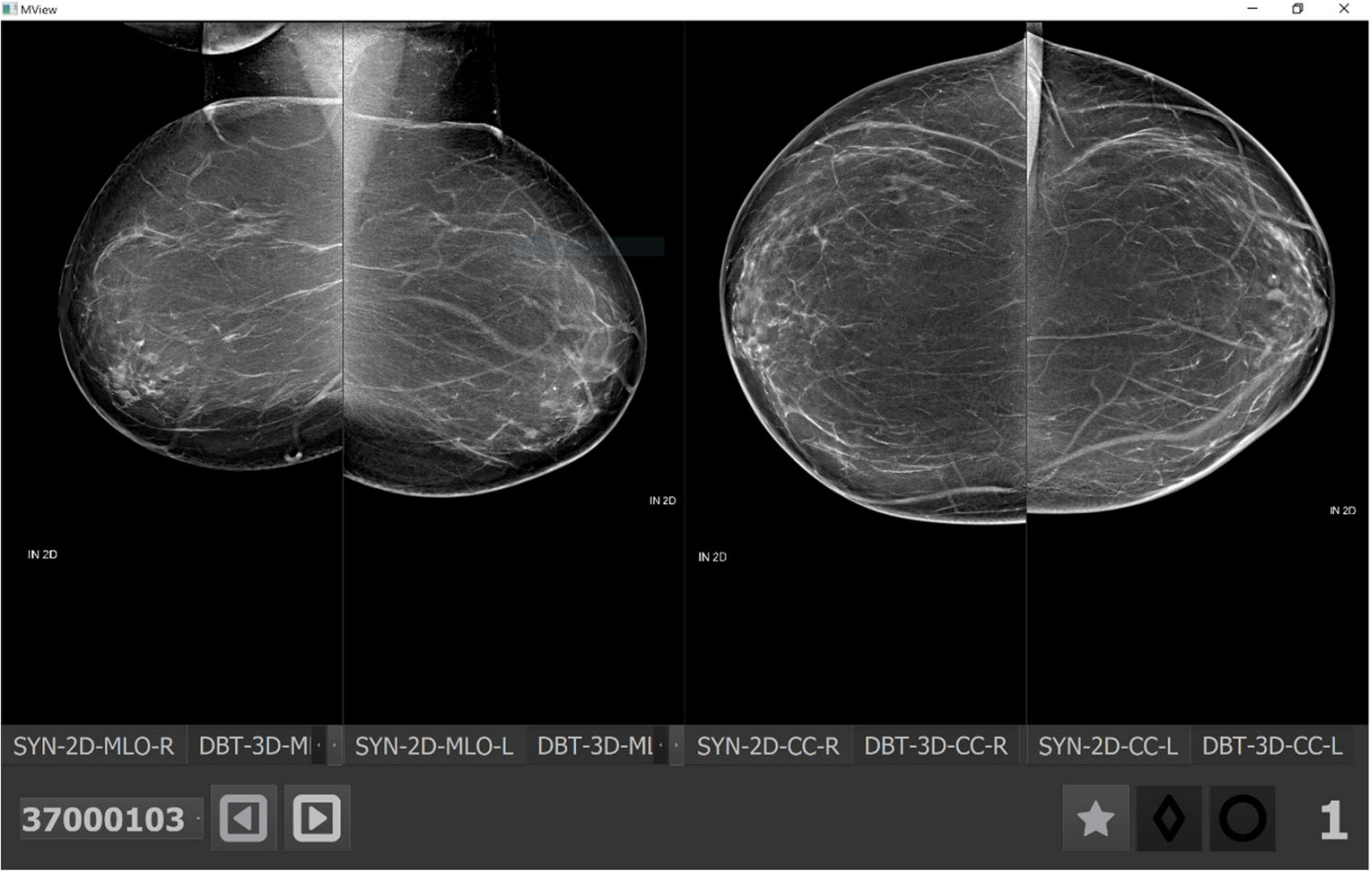
Breast tomosynthesis exam (the synthetic image) of a woman without cancer and an exam-level cancer likelihood score of 1 (lowest) by the AI system. When reading the case aided, 17/18 (94%) radiologists read the exam faster, with an average reduction of reading time of −54% (from 36 to 19 s)

The reading time reduction using AI support was correlated with the exam-level score assigned by the AI system: reductions were stronger for the lowest exam-level scores (−30%) (see Fig. 3). Figure 4 shows an example where radiologists consistently read the exam faster when using AI support.

### Impact on sensitivity and specificity

**Fig. 5:**
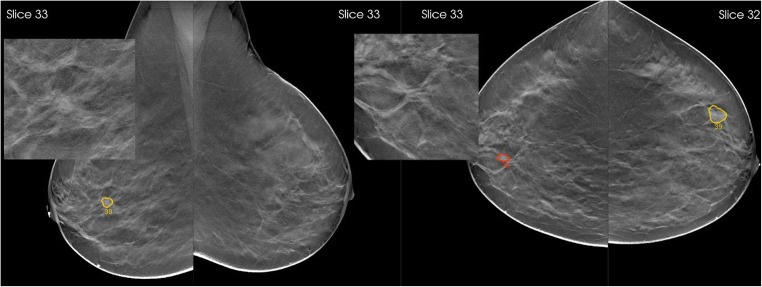
Breast tomosynthesis exam of a woman with an architectural distortion in the right breast, proven to be a 15-mm invasive ductal carcinoma (zoomed). The AI system marked the regions and assigned region-scores of 76 and 39 on cranio-caudal and mediolateral oblique views, respectively, and an exam-level cancer likelihood score of 10, the highest category. When reading the case unaided, 8/18 (44%) radiologists would have recalled the woman, a proportion that increased to 15/18 (83%) radiologists when reading the case with AI support

Using AI support, radiologists significantly improved their cancer detection sensitivity. The average sensitivity increased from 74.6 (95% CI = 68.3–80.8%) to 79.2% (95% CI = 73.3–85.1%), a relative difference of +6.2% (95% CI = 1.3–11.1%), *p* = 0.016. Specificity was maintained: a relative difference of + 1.1% (95% CI = −1.3%, 3.5%), *p* = 0.380. Figure 5 shows an example where radiologists consistently improved sensitivity when using AI support.

### Stand-alone AI detection performance

**Fig. 6:**
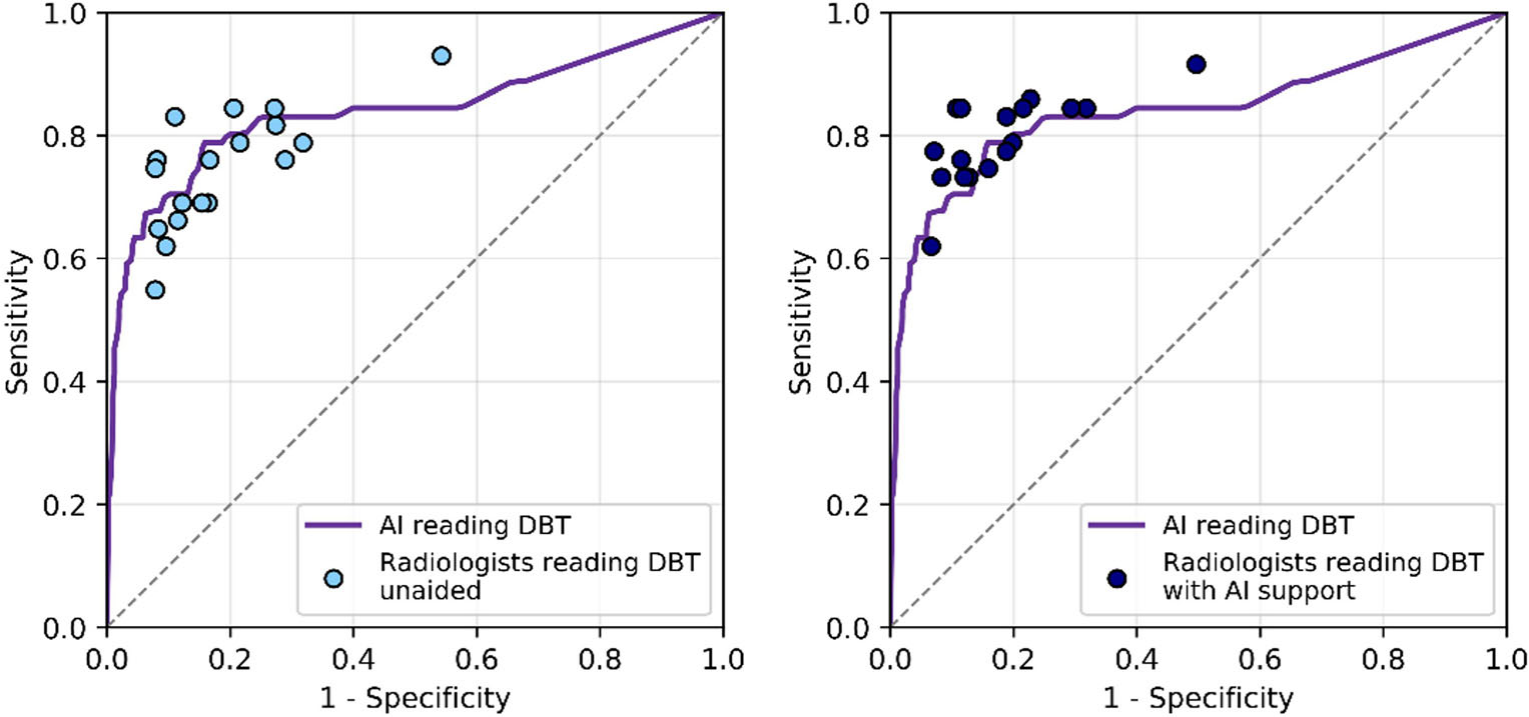
Stand-alone receiver operating characteristic curve of the AI support system, together with the operating points of the 18 individual radiologists reading breast tomosynthesis (DBT) unaided (left) or with AI support (right)

The stand-alone AUC of the AI system was 0.840 (SE = 0.034), +0.007 higher (95% CI = −0.048, 0.062) compared to the average unaided radiologist AUC. This performance was statistically non-inferior (*p* = 0.8115). Descriptively, the stand-alone AUC was higher compared to the AUC of 10/18 radiologists, reading DBT unaided. The stand-alone ROC curve of the AI system compared to radiologists’ performance is depicted in Fig. 6.

## Discussion

This study shows that a deep learning–based AI system for DBT enables radiologists to increase their breast cancer detection performance in terms of overall accuracy and sensitivity at similar specificity, while reducing reading time. The observed improvement in accuracy is comparable to what has been reported with an earlier version of this AI program for mammography evaluation and another AI program for evaluation of narrow-angle DBT [10, 16]. The sensitivity improvement could help to reduce the number of DBT screening false negatives, i.e. lesions being overlooked or misdiagnosed [3], while the reduced reading time might enable the implementation of DBT for screening in sites where DBT is currently considered too time-intensive. Furthermore, the improvement in AUC was observed for all radiologists regardless of the time they dedicate to breast imaging in clinical practice.

The reduction in reading time per DBT exam when concurrently using an AI system for reading support is similar to those from other studies [16, 17]. Nevertheless, reading times heavily rely on the specific functionality of the viewing application used for interpretation, as well as the exact viewing protocol used to evaluate the DBT exams, which prevents direct comparisons among the available studies. For example, the average unaided reading time in the study by Conant et al [16] using another AI software was almost twice as long as the one found in our study, but interestingly, the AI-assisted reading times were similar in both studies; approximately 30 s per four-view DBT exam with 2D synthetic images. Also, observing the largest reading time reduction in this study for the readers presented with an SM for navigation suggests that functionality of the reading environment is an important factor.

Similar to results in a previous 2D mammography study with this AI system [10], a strong dependency of the reading time reduction on the exam-based AI scores was found (Fig. 3). This suggests that the biggest reading time reduction can be achieved for the lowest exam-based AI scores, indicating the readers were confident enough to spend less time on exams categorized as most likely normal, despite the relatively short time to get familiar with the AI system. The resulting reading times per category may be used to estimate the potential of AI support in a representative series of screening exams. Provided that readers will be using 2D SM images and interactive navigation, the reading time reduction in a screening population would be approximately −20% (95% CI = −16%, −25%).

The increased accuracy and decreased reading time were observed using cases from a previously, prospectively collected dataset, consisting of DBT exams obtained with a large acquisition angle. Since a previous study [10] showed similar improvements for 2D mammography, it is likely that the AI induced improvements hold true for the whole spectrum of mammographic techniques, which is corroborated by the fact that the stand-alone performances of the AI system equal the radiologists for both wide-angle DBT and 2D mammography.

As the stand-alone performance of the AI system was equivalent to the performance of the radiologists, it may be feasible to explore implementation strategies beyond the concurrent reading of DBT exams with AI support. Like in 2D mammography, it might be feasible to use AI for efficient triaging of the screening workload [14, 15]. By identifying a large group of normal exams with a high negative predictive value, alternative strategies such as single-reading when double-reading, or even exclusion from radiologist evaluation, could be explored.

A limitation of this study is the use of a cancer-enriched dataset instead of a consecutively collected sample of screening mammograms from a clinical setting. This was to allow a multi-reader evaluation with sufficient findings to draw useful conclusions. Consequently, this may not be fully representative of a real screening situation. To what extent this difference affects the results is unknown. The effectiveness of AI support in screening with DBT still needs to be assessed: results from this study should serve as a starting point for prospective studies focusing on the impact of using AI in DBT mammography in clinical and screening environments. In conclusion, radiologists improved their cancer detection in DBT examinations when using an AI support system, while simultaneously reducing reading time. Using an AI reading support system could allow for more cost-effective screening programs with DBT.

## Abbreviations

AI: Artificial intelligence
AUC: Area under the ROC curve
CC: Cranio-caudal
CI: Confidence interval
DBT: Digital breast tomosynthesis
GLME: Generalized linear mixed-effects
MLO: Mediolateral oblique
MRMC: Multi-reader multi-case
ROC: Receiver operating characteristic
SM: Synthetic mammography

## Declarations

### Guarantor

The scientific guarantor of this publication is Ritse M. Mann, PhD, MD.

### Statistics and biometry

One of the authors has significant statistical expertise.

The tomosynthesis image files are owned by a third company party (Siemens Medical Solutions, USA) and were made available for research purposes to ScreenPoint Medical (Nijmegen, The Netherlands) only. Without the DBT image data, the observer study data alone is of limited value and challenging to interpret. However, if needed, the observer study data could be made available for auditing purposes.

### Methodology

• Prospective

• Diagnostic or prognostic study

• Multicenter study

## References

[1] Rafferty EA, Durand MA, Conant EF (2016). Breast cancer screening using tomosynthesis and digital mammography in dense and nondense breasts. JAMA..

[2] Friedewald SM, Rafferty EA, Rose SL (2014). Breast cancer screening using tomosynthesis in combination with digital mammography. JAMA..

[3] Zackrisson S, Lång K, Rosso A (2018). One-view breast tomosynthesis versus two-view mammography in the Malmö Breast Tomosynthesis Screening Trial (MBTST): a prospective, population-based, diagnostic accuracy study. Lancet Oncol.

[4] Bernardi D, Gentilini MA, De Nisi M (2019). Effect of implementing digital breast tomosynthesis (DBT) instead of mammography on population screening outcomes including interval cancer rates: Results of the Trento DBT pilot evaluation. Breast.

[5] Sechopoulos I (2013). A review of breast tomosynthesis. Part I. The image acquisition process. Med Phys.

[6] Dang PA, Freer PE, Humphrey KL, Halpern EF, Rafferty EA (2014). Addition of tomosynthesis to conventional digital mammography: effect on image interpretation time of screening examinations. Radiology..

[7] Rodriguez-Ruiz A, Gubern-Merida A, Imhof-Tas M (2017). One-view digital breast tomosynthesis as a stand-alone modality for breast cancer detection: do we need more?. Eur Radiol.

[8] Rimmer A (2017) Radiologist shortage leaves patient care at risk, warns royal college. BMJ 359. 10.1136/bmj.j4683 10.1136/bmj.j4683 29021184

[9] Litjens G, Kooi T, Bejnordi BE, Setio AAA, Ciompi F (2017). A survey on deep learning in medical image analysis. Med Image Anal.

[10] Rodríguez-Ruiz A, Krupinski E, Mordang J-J, Schilling K (2018). Detection of breast cancer with mammography: effect of an artificial intelligence support system. Radiology..

[11] Rodriguez-Ruiz A, Lång K, Gubern-Merida A, Broeders M et al (2019) Stand-alone artificial intelligence for breast cancer detection in mammography: comparison with 101 radiologists. J Natl Cancer Inst 111(9) 10.1093/jnci/djy222 PMC6748773 30834436

[12] Wu N, Phang J, Park J (2019). Deep neural networks improve radiologists’ performance in breast cancer screening. IEEE Trans Med Imaging.

[13] McKinney SM, Sieniek M, Godbole V (2020). International evaluation of an AI system for breast cancer screening. Nature..

[14] Yala A, Schuster T, Miles R, Barzilay R, Lehman C (2019). A deep learning model to triage screening mammograms: a simulation study. Radiology..

[15] Rodriguez-Ruiz A, Lång K, Gubern-Merida A, Teuwen J (2019). Can we reduce the workload of mammographic screening by automatic identification of normal exams with artificial intelligence? A feasibility study. Eur Radiol.

[16] Conant EF, Toledano AY, Periaswamy S (2019). Improving accuracy and efficiency with concurrent use of artificial intelligence for digital breast tomosynthesis. Radiol Artif Intell.

[17] Chae EY, Kim HH, Jeong J-w, Chae S-H, Lee S, Choi Y-W (2018). Decrease in interpretation time for both novice and experienced readers using a concurrent computer-aided detection system for digital breast tomosynthesis. Eur Radiol.

[18] Rodriguez-Ruiz A, Castillo M, Garayoa J, Chevalier M (2016). Evaluation of the technical performance of three different commercial digital breast tomosynthesis systems in the clinical environment. Phys Med.

[19] Georgian-Smith D, Obuchowski NA, Lo JY et al (2019) Can digital breast tomosynthesis replace full-field digital mammography? A multireader, multicase study of wide-angle tomosynthesis. AJR Am J Roentgenol. 212(6):1393–1399 10.2214/AJR.18.20294 30933648

[20] Siemens Medical Solutions USA Inc. (2015) FDA application: mammomat inspiration with digital breast tomosynthesis. https://www.accessdata.fda.gov/cdrh_docs/pdf14/P140011b.pdf

[21] Rodriguez-Ruiz A, Teuwen J, Vreemann S, Bouwman RW et al (2017) New reconstruction algorithm for digital breast tomosynthesis: better image quality for humans and computers. Acta Radiol 284185117748487 10.1177/0284185117748487 PMC6088454 29254355

[22] Hillis SL, Obuchowski NA, Berbaum KS (2011). Power estimation for multireader ROC methods: an updated and unified approach. Academic Radiology..

[23] Kooi T, Litjens G, van Ginneken B (2017). Large scale deep learning for computer aided detection of mammographic lesions. Med Image Anal.

[24] Mordang J-J, Janssen T, Bria A, Kooi T, Gubern-Mérida A, Karssemeijer N (2016) Automatic microcalcification detection in multi-vendor mammography using convolutional neural networks. International Workshop on Digital Mammography. Springer. 9699:35–42

[25] Tabata K, Uraoka N, Benhamida J (2019). Validation of mitotic cell quantification via microscopy and multiple whole-slide scanners. Diagn Pathol..

[26] Obuchowski NA (1997) Nonparametric analysis of clustered ROC curve data. Biometrics 567-78 9192452

[27] Obuchowski NA (1995). Multireader, multimodality receiver operating characteristic curve studies: hypothesis testing and sample size estimation using an analysis of variance approach with dependent observations. Acad Radiol.

[28] McCullagh P (2019) Generalized linear models. Routledge

[29] Gallas B (2017) iMRMC v4.0: Application for analyzing and sizing MRMC reader studies. https://github.com/DIDSR/iMRMC/releases, https://cran.r-project.org/web/packages/iMRMC/index.html

